# Anti-Oxidative and Anti-Apoptotic Effects of Apigenin on Number of Viable and Apoptotic Blastomeres, Zona Pellucida Thickness and Hatching Rate of Mouse Embryos

**DOI:** 10.22074/ijfs.2018.5392

**Published:** 2018-06-20

**Authors:** Manouchehr Safari, Houman Parsaie, Hamid Reza Sameni, Mohammad Reza Aldaghi, Sam Zarbakhsh

**Affiliations:** Nervous System Stem Cells Research Center, Faculty of Medicine, Semnan University of Medical Sciences, Semnan, Iran

**Keywords:** Apigenin, Apoptosis, Blastomeres, Embryonic Development, Zona Pellucida

## Abstract

**Background:**

Apigenin is a plant-derived compound belonging to the flavonoids category and bears protective effects on different cells. The aim of this study was to evaluate the effect of apigenin on the number of viable and apoptotic blastomeres, the zona pellucida (ZP) thickness and hatching rate of pre-implantation mouse embryos exposed to H_2_O_2_ and actinomycin D.

**Materials and Methods:**

In this experimental study, 420 two-cell embryos were randomly divided into six groups: i. Control, ii. Apigenin, iii. H_2_O_2_ , iv. Apigenin+H_2_O_2_ , v. Actinomycin D, and vi. Apigenin+Actinomycin D. The percentage of blastocysts and hatched blastocysts was calculated. Blastocyst ZP thickness was also measured. In addition, viable blastomeres quantity was counted by Hoechst and propidium iodide staining and the number of apoptotic blastomeres was counted by TUNEL assay.

**Results:**

The results of viable and apoptotic blastomeres quantity, the ZP thickness, and the percentage of blastocysts and hatched blastocysts were significantly more favorable in the apigenin group, rather than the control group (P<0.05). The results of the apigenin+H_2_O_2_ group were significantly more favorable than the H_2_O_2_ group (P<0.05); and the results of apigenin+actinomycin D group were significantly more favorable than actinomycin D group (P<0.05).

**Conclusion:**

The results suggest that apigenin may protect mouse embryos against H_2_O_2_ and actinomycin D. So that it increases the number of viable blastomeres and decreases the number of apoptotic blastomeres, which may cause expanding the blastocysts, thinning of the ZP thickness and increasing the rate of hatching in mouse embryos.

## Introduction

Embryonic development in culture medium may be affected by several stressors such as high oxygen concentration and high level of reactive oxygen species (ROS) (1). It is important that embryos in culture media to be protected from oxidative stress. For this purpose, antioxidants are valuable candidates (2).

Apigenin is a plant-derived compound belonging to the flavonoids category presented in various fruits and vegetables, such as parsley, onion, celery, chamomile and orange (3). Apigenin has different biological activities such as anti-oxidative, anti-inflammatory, anti-cancer and anti-tumorigenic properties (4, 5). Apigenin protects DNA from oxidative stress by binding to nucleic acids. Moreover, apigenin prevents cell apoptosis by suppressing ROS compounds (6) and decreasing the expression of caspase-3, caspase-9 and TNF-α (7).

By studying the embryo morphology, prediction of embryo fate is largely possible. The most morphological indicators to select the best embryos for transferring are zona pellucida (ZP) thickness and blastomere quantity (8). ZP thickness is a reliable indicator of *in vitro* fertilization (IVF) success rate which can be applied as a criterion for embryo selection. Actually, ZP thickness is inversely correlated with embryo viability and hatching rate (9). Moreover, cleavage rate and development to blastocyst are applied as two quality parameters of mammal embryos (8).

Although the beneficial effects of apigenin on different cells and tissues have been investigated (10, 11), there is no report yet concerning the effect of apigenin on growth and quality of embryos. So, in this study, we evaluated for the first time the impact of apigenin on some morphological indicators of pre-implantation mouse embryos including ZP thickness, viable and apoptotic blastomere quantity and hatching rate. To evaluate the anti-oxidant and anti-apoptotic effects of apigenin, we used H_2_O_2_ and actinomycin D in the culture medium to create ROS and apoptosis.

## Materials and Methods

In this experimental study, female C57BL/6 mice (6-8 weeks) were kept under controlled temperature (25 ± 2°C) and light (12 hours light/12 hours dark), with free access to food and water. All animal protocols were approved by the Research Council of Semnan University of Medical Sciences (Semnan, Iran).

### Superovulation and embryo collection

For superovulation, the mice received 10 IU pregnant mare's serum gonadotropin (PMSG, Sigma, China) intraperitoneally. 48 hours later, they received 10 IU human chorionic gonadotropin (hCG, Sigma, China) intraperitoneally (12). They were subsequently mated overnight with males and the mating was assessed by the presence of vaginal plug on the morning after hCG injection. Two-cell embryos were flushed from the oviduct at about 48 hours after hCG injection and washed in human tubal fluid (HTF) medium containing HEPES (Sigma, USA). A total of 420 two-cell embryos were used in this study.

### Embryo culture

The embryos were transferred into the HTF medium, supplemented with 10% human serum albumin (Sigma, USA). Two-cell embryos were randomly divided into six groups (70 embryos in each group): i. Control group, without any treatment, ii. Apigenin (Sigma, China) group, 10 µM apigenin was added into the medium, iii. H_2_O_2_ group, 500 µM H_2_O_2_ was added into the medium, iv. Apigenin+H_2_O_2_ group, 10 µM apigenin and 500 µM H_2_O_2_ were added into the medium, v. Actinomycin D (Sigma, USA) group, 0.005 µg/ml actinomycin D was added into the medium, vi. Apigenin+actinomycin D group, 10 µM apigenin and 0.005 µg/ml actinomycin D were added into the medium. In all groups, 10 embryos were placed in a drop (20 µl) of HTF medium under mineral oil (Sigma, USA) in a 35 mm Petri dish (Jet Biofil, Canada). Next, they were incubated at 37°C with 95% humidity and 5% CO2. To evaluate the antioxidant effect of apigenin, two- to four-cell embryos were exposed to 500 µM H_2_O_2_ in the culture medium for 72 hours. To evaluate the anti-apoptotic effect of apigenin, as soon as reaching two-cell embryos to eight-cell stage, they were incubated with 0.005 µg/ml actinomycin D in the medium for 4 hours (13). Eventually, on the fourth and fifth days of embryonic period, the percentage of embryos reaching the stages of blastocyst and hatched blastocyst was assessed (14).

### Measurement of zona pellucida thickness

To measure ZP thickness, the blastocysts were randomly selected. Measurement was taken from the images using an inverted microscope (Nikon, Eclipse Ti-U, Japan) and motic images plus 2.0 software. The thickness of each ZP was measured at three points (8, 15).

### Differential staining and TUNEL assay

The blastocysts were randomly selected for blastomere counting analysis. Differential staining of blastocysts and apoptotic nuclei detection were performed according to the method described by Fouladi-Nashta et al. (16, 17). The blastocysts were treated with 30 µg/ ml propidium iodide (PI, Sigma, China) and 1% Triton X-100 (Sigma, China) at 37°C for 5 minutes. Immediately after, the blastocysts were washed twice and fixed in 4% paraformaldehyde containing 10 µg/ml bisbenzimide (Hoechst 33342, Sigma, USA) for 20 minutes at room temperature leading to fixation of blastocysts and staining total cell nuclei. Next, embryos were washed and incubated in droplets of in situ cell death detection (TUNEL) kit solution (Roche, Germany) for 45 minutes according to the manufacturer’s instructions. Then the embryos were mounted on glass slides in glycerol droplets and were observed under a fluorescent microscope (Motic, AE31, Spain). Trophectoderm (TE) nuclei labeled with PI were appeared red, total cells including inner cell mass (ICM) labeled with Hoechst were appeared blue and apoptotic cells labeled with TUNEL were appeared green. The number of ICM, TE, and apoptotic cells was counted.

### Statistical analysis

Statistical analysis was performed using SPSS software version 16.0 software (version 16.0 for windows, Chicago, IL, USA). Comparison of the percentage of embryos from two-cell to hatched blastocyst was analyzed by x2 test. The results of embryo percentage in apigenin group were compared to control group, the results of apigenin+H_2_O_2_ group were compared to H_2_O_2_ group, and the results of apigenin+actinomycin D group were compared to actinomycin D group. The results of ZP thickness and number of viable and apoptotic blastomeres were analyzed by one-way ANOVA followed by the Tukey test. The results are presented as mean ± SEM. P<0.05 is considered statistically significant.

## Results

### Developmental rate of embryos

**Fig.1:**
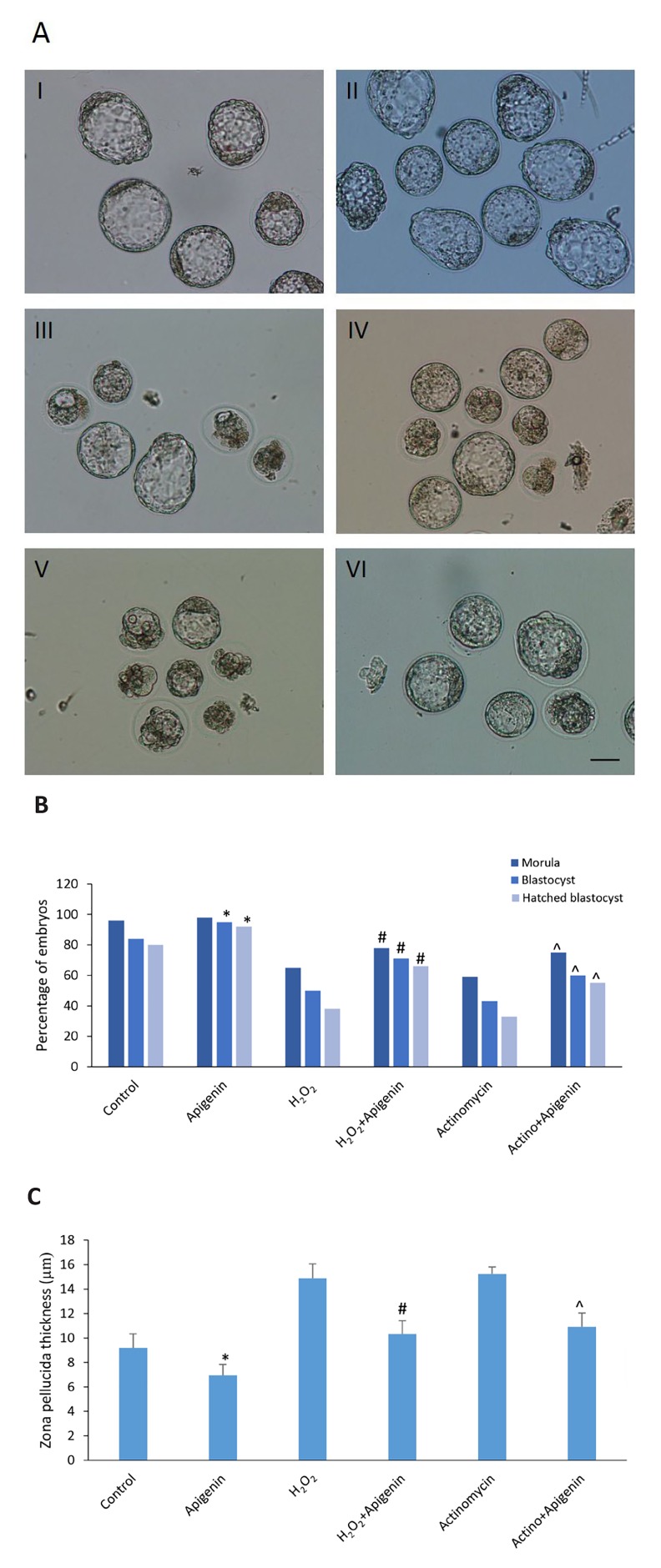
Apigenin protected the embryos against H_2_O_2_ and actinomycin D. A. The blastocysts of I. Control group, II. Apigenin group, III. H_2_O_2_ group, IV.Apigenin+H_2_O_2_ group, V. Actinomycin D group, VI. Apigenin+actinomycin D group, B. The results of the percentage of embryos that have reached to the stages of morula, blastocyst and hatched blastocyst, and C. The results of zona pellucida thickness of blastocysts (scale bar: 50 µm). Values are presented as mean ± SEM. *; P<0.05 apigenin versus the control group, #; P<0.05 apigenin+H_2_O_2_ versus the H_2_O_2_ group, and ^; P<0.05 apigenin+actinomycin D versus the actinomycin D group.

There was no statistically significant difference in the percentage of two-cell embryo development to eight- cell between the control and apigenin groups (P=0.404). There was no statistically significant difference between apigenin+H_2_O_2_ group and H_2_O_2_ (P=0.382). In addition, no statistically significant difference was determined between the apigenin+actinomycin D group and the actinomycin D (P=0.466, Table 1). Percentage of embryos reached to morula stage in the apigenin+H_2_O_2_ group was significantly higher than the H_2_O_2_ group (P=0.037), and in the apigenin+actinomycin D group was significantly higher than the actinomycin D group (P=0.016). There was no statistically significant difference in morula stage between the apigenin and control groups (P=0.087). Percentage of embryos reached to blastocyst and hatched blastocyst stages in the apigenin group was significantly higher than the control group (P=0.022). Additionally, this was significantly higher in the apigenin+H_2_O_2_ compared to the H_2_O_2_ group (P<0.001), and in apigenin+actinomycin D group compared to actinomycin D group (P<0.001, Fig .1A, B).

### The zona pellucida thickness of blastocysts

The results showed that ZP thickness of blastocysts in the apigenin group was significantly thinner than the control group (P=0.034). It was significantly thinner in the apigenin+H_2_O_2_ group compared to the H_2_O_2_ group (P=0.023), and in the apigenin+actinomycin D group rather than the actinomycin D group (P=0.003, Fig .1A, C).

### Viable blastomeres quantity

**Fig.2:**
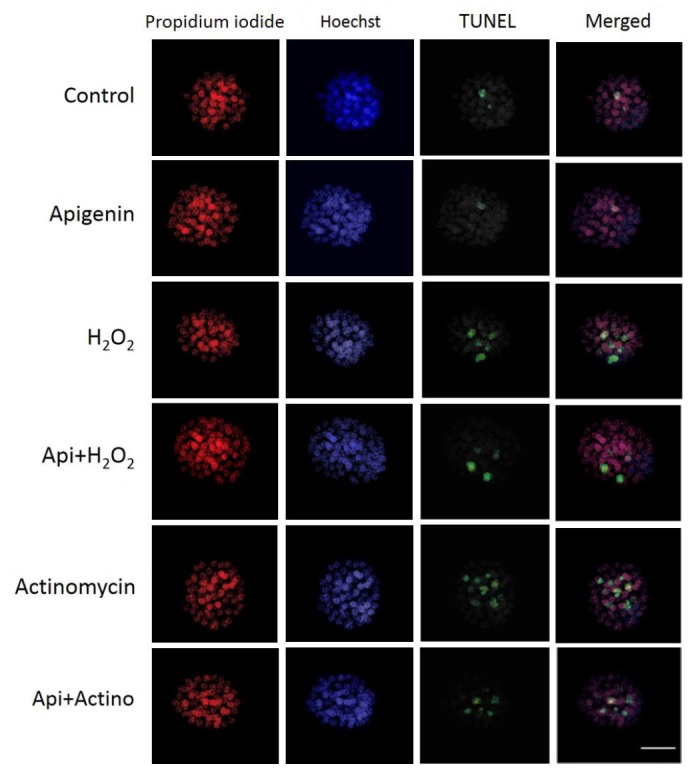
Differential staining and TUNEL labeling of the blastomeres. Staining with propidium iodide for trophectoderm cells (red), Hoechst for total cells (blue), and TUNEL for apoptotic cells (green) (scale bar: 50 µm).

**Fig.3:**
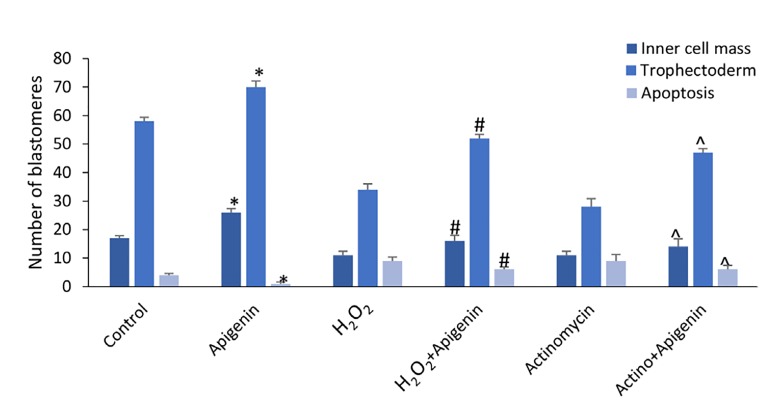
The results of viable blastomeres with propidium iodide and Hoechst staining and the apoptotic blastomeres with TUNEL assay. Values are presented as mean ± SEM. *; P<0.05 apigenin versus the control group, #; P<0.05 apigenin+H_2_O_2_ versus the H_2_O_2_ group, and ^; P<0.05 apigenin+actinomycin D versus the actinomycin D group.

The blastocysts were stained with Hoechst and PI followed by quantifying ICM and TE (Fig .2). The results showed the number of ICM and TE in the apigenin group was significantly higher than the control group (P=0.037). In addition, it was significantly higher in the apigenin+H_2_O_2_ group compared to the H_2_O_2_ group (P<0.001) and in the apigenin+actinomycin D group rather than the actinomycin D group (P<0.001, Fig .3). 

### Apoptotic blastomeres quantity

The apoptotic blastomeres were detected by TUNEL assay (Fig .2). The results showed that apoptotic blastomeres quantity in the apigenin group was significantly lower than the control group (P=0.011). Similarly this number was significantly lower in the apigenin+H_2_O_2_ group compared to the H_2_O_2_ group (P=0.003), and in the apigenin+actinomycin D group rather than actinomycin D group (P<0.001, Fig .3). 

**Table 1:** The results of number and percentage of two-cell embryos to eight-cell embryos in all groups


Group	2-Cell (%)	4-Cell (%)	8-Cell (%)

Control	70 (100)	68 (97.1)	66 (94.2)
Apigenin	70 (100)	69 (98.6)	68 (97.1)
H_2_O_2_	70 (100)	66 (94.2)	62 (88.5)
Apigenin+H_2_O_2_	70 (100)	68 (97.1)	65 (92.8)
Actinomycin D	70 (100)	67 (95.7)	65 (92.8)
Apigenin+Actinomycin D	70 (100)	68 (97.1)	67 (95.7)


## Discussion

During development of embryos *in vitro*, various harmful factors can affect embryo quality and fertilization, although it seems that anti-oxidants can reduce the amount of damage (2, 18). In this study, for the first time, we evaluated the effect of apigenin on some morphological indicators of pre-implantation mouse embryos including ZP thickness, number of viable and apoptotic blastomeres and hatching rate. Moreover, to evaluate the anti-oxidant and anti-apoptotic effects of apigenin, we used H_2_O_2_ and actinomycin D in the culture medium to induce oxidative stress and apoptosis. Overall, the results showed that 10 µM apigenin, by protecting the embryos against H_2_O_2_ and actinomycin D, was able to enhance the quality and development of embryos and reduce apoptosis in the blastomeres.

Anti-oxidant capacity of apigenin has been shown in different cell types. So that Zhang et al. (19) reported that apigenin has neuro-protective effect on rats after contusive spinal cord injury. Zhao et al. (20) reported that apigenin has neuro-protective, anti-amyloidogenic and neuro-trophic effects on an Alzheimer disease mouse model. Liu et al. (21) reported that apigenin expresses Oct-4, Sox2, and c-Myc in dental pulp cells which helps maintain the dental pulp cells in an undifferentiated stage. However, no report concerns the effect of apigenin on growth and quality of embryos.

In the present study, to evaluate the anti-oxidant effect of apigenin, H_2_O_2_ was used, which similar to ROS easily penetrates from the cell membrane, causing damage and apoptosis (22). Sharma et al. (6) reported that apigenin attaches to nucleic acid bases and decreases oxidative DNA damage in epithelial cells of prostate. Lagoa et al. (23) showed that flavonoids including apigenin inhibit H_2_O_2_ production by increasing mitochondrial activity. The purpose of exposing embryos to H_2_O_2_ was to exacerbate the conditions of ROS in the culture medium (24) and evaluate the anti-oxidant effect of apigenin on protection of the embryos. The results of present study showed that apigenin by reducing the effects of H_2_O_2_ could protect the embryos and improve embryonic development. These results were in agreement with the other related study (25).

Moreover, to evaluate the anti-apoptotic effect of apigenin, actinomycin D was used as an inducer of apoptosis on different cell types by connecting to guanine-cytosine base pairs and inhibiting DNA transcription (26). Niknafs et al. (27) showed that melatonin improved development of the early mouse embryos exposed to actinomycin D. Abdelrazik et al. (13) reported that l-carnitine reduces apoptosis rate in blastomeres of mouse embryos exposed to actinomycin D. The results of present study showed that apigenin could protect the embryos exposed to actinomycin D and decrease the rate of apoptosis. These results were in agreement with other related studies (13, 27, 28).

Embryo quality is evaluated with morphological parameters. Viable and apoptotic blastomeres quantity, ZP thickness and ability to hatch of blastocyst are some of the most important morphological parameters of embryo (29, 30). Various studies have reported that reducing number of blastomeres could decrease chance of survival of embryos (31, 32). While the number of blastomeres increase, ZP thickness is decreased; in contrast the probability of blastocyst hatching and successful implantation are increased (15, 33).

Regarding the anti-oxidant and anti-apoptotic properties of apigenin, protective effect of this agent on improvement embryo growth is probably due to reduction of the ROS level (34), maintaining the mitochondrial activity (11, 35) and upregulating the gene expression of anti-oxidant enzymes like glutathione peroxidase (25). Glutathione peroxidase is an enzymatic anti-oxidant expressing in many cells and tissues during embryo formation and protecting the embryo against oxidative stress (18, 36). Since the glutathione peroxidase removes H_2_O_2_ , apoptosis in embryonic cells reduces (18). Han et al. (37) reported that apigenin reduces oxidative stress and neuronal apoptosis in early brain injury following subarachnoid hemorrhage.

ZP thickness is a marker to select the best frozen-thawed embryos for transfer (38), because thin ZP increases the probability of hatching rate and implantation. ZP thickness depends on inherent features of embryos to generate the lytic factors needed for ZP thinning (9, 39). There are many ways to thin or remove ZP such as partial zona dissection, using proteolytic enzymes, laser and Tyrode’s solution (2). But those methods are invasive regarding that adding anti-oxidant into the embryo culture medium is probably less invasive and may cause thinning ZP thickness (15, 40). The present study showed apigenin could decrease ZP thickness of blastocysts. These results are in agreement with the results of Khanmohammadi et al. (15) indicating that l-carnitine, as an antioxidant, has the ability to reduce ZP thickness.

Despite obtaining these results, there are some limitations in this study. More research is required to clarify the molecular mechanisms underlying apigenin function on development and qualifying embryos. In addition, the number of samples was low. Hence, more samples would be needed in different conditions and with different doses of apigenin.

## Conclusion

The results of this study suggest that apigenin with anti- oxidant and anti-apoptotic properties may protect the embryos against H_2_O_2_ and actinomycin D. Apigenin can probably increase the number of viable blastomeres and decrease the number of apoptotic blastomeres, which may cause expanding blastocysts, thinning ZP thickness and increasing the rate of hatching in mouse embryos.
